# Breakfast Time Blackouts

**DOI:** 10.1155/2014/568983

**Published:** 2014-09-18

**Authors:** Andrew W. Barritt, Bridget K. MacDonald

**Affiliations:** ^1^Hurstwood Park Neurosciences Centre, Haywards Heath, West Sussex RH16 4EX, UK; ^2^Department of Neurology, St George's Hospital, Tooting, London SW17 0QT, UK

## Abstract

We present the case of a 16-year-old girl who suffered from repeated episodes of collapse and loss of consciousness which could be provoked by undertaking a stretching manoeuvre comprising a combined breath hold and neck torsion. A review of the literature is provided on other cases of so-called “stretch syncope” which appears to be a rare form of reflex syncope affecting patients in adolescence.

## 1. Case History

A 16-year-old female college student presented with episodes of sudden onset collapse over the previous seven weeks associated with a brief loss of consciousness lasting from three to eight seconds. The attacks occurred soon after waking in the morning and always from a standing position. Family members described her appearance as pale throughout. However, she was not seen to make additional rhythmic movements, change her respiratory pattern, sweat noticeably, bite her tongue or become incontinent. She would be rapidly orientated afterwards but could not remember the period of collapse itself. There was no significant past medical history, including hypermobility syndrome, she was on no regular medications, and none of her relatives had ever been affected. General examination of the cardiovascular, respiratory, and abdominal systems was normal, with a regular pulse at 80 beats per minute, supine blood pressure of 110/70 mmHg, and no postural drop after 3-minute standing. Routine blood tests were normal including fasting blood glucose of 5 mmol/L. An electrocardiogram demonstrated normal sinus rhythm, a urine dip was unremarkable, and a routine AP chest radiograph showed clear lung fields and a normal cardiac contour. On closer questioning it became apparent that each episode was preceded by a yawn-stretch manoeuvre whereby yawning was simultaneously accompanied by flexion, abduction, and external rotation of both arms, neck extension with lateral rotation, and arching backwards of her trunk in a hyperlordosis. Loss of consciousness would then ensue within three to four seconds and the episode would proceed as above. The phenomenon could thereby be reproduced voluntarily, although a yawn without stretching was insufficient to elicit her collapses.

## 2. Discussion

Syncope induced by stretching is a recognised, but rarely reported, phenomenon in adolescents. To the authors' knowledge, there have been only thirteen other cases described in the literature and, of these, the majority have been adolescent males [1–5]. However, the precise pathophysiology is unclear and both direct (vertebral) arterial compression [1, 2, 4] and reflex mechanisms [3, 5] have been proposed.

A typical “stretch” which encompasses back hyperextension, shoulder abduction, and neck extension has long been considered to include a straining phase against a closed glottis (or Valsalva manoeuvre). Sharpey-Schafer recognised back in 1965 that such posturing in “young males who leap out of bed, stand on tiptoe, and stretch yawn” could provoke loss of consciousness [6]. It is, however, worth noting the suggestion that a yawn-stretch manoeuvre may not incorporate a typical Valsalva strain owing to the relative predominance of the slow maximal inspiration and expiration of the yawn [7]. Nevertheless, patients affected by syncope induced purely by stretching, so-called stretch syncope in adolescence (SSA), have described feelings of light-headedness, visual blurring, occipital headache, depersonalization, and even déjà vu precipitated within seconds by a stretch even without yawning and preceding a frank loss of consciousness [1–4]. In some instances, these “presyncopal” symptoms have been terminated and a collapse averted, by reassuming a forward neck flexion [1, 4]. In addition, several patients have been seen to have occasional muscle twitches affecting the head and upper limbs either prior to or during the fall to the ground, and one patient only ever suffered brief periods of altered awareness rather than a frank loss of consciousness prompting prolonged, yet unsuccessful, treatment with antiepileptic medication [3]. The case series by Pelekanos and colleagues recognised that a Valsalva manoeuvre alone was insufficient to induce the patients' syncope, which has been corroborated in subsequent cases [1, 4, 5], and that simultaneous hyperextended head posturing was required [2]. Indeed, it was first hypothesised that this posturing caused warping of the posterior cervical tissues and subsequent obstruction to blood flow within the vertebral arteries, thereby causing vertebrobasilar ischaemia.

Sturzenegger and colleagues reported two male cases of SSA and used Doppler ultrasound over the temporal bone “window” to monitor blood flow within their posterior cerebral arteries (PCAs) during a selection of head manoeuvres along with concurrent arm flexion and shoulder hyperabduction. Only the combination of neck extension and the outstretched arm posturing was seen to precipitate a significant decreased flow of blood with presyncopal symptoms being reported about 4 seconds later [4]. A transient reactive hyperaemia was then seen roughly 15 seconds after returning the head to a neutral forward position. Baseline imaging tests on these patients, including brain and cervical spine MRI, cervical radiographs, extracranial Doppler ultrasound, and four vessel catheter angiogram, were normal. Attempts to reproduce the stretching steps during the angiogram were technically difficult and abandoned. Mazzuca and Thomas [1] subsequently described a male case who complained of either visual flashes or collapses preceded by stretching and to whom they subjected the same combinations of head and arm posturing mentioned above. He reported his visual flashes within five seconds of neck hyperextension and arm abduction/extension. Decreased blood flow within the PCAs on transcranial Doppler was, similarly, demonstrated along with the reactive hyperaemia and tachycardia 15 seconds after reassuming a neutral head position. Electroencephalography and electrocardiography were also performed during these episodes: low amplitude QRS complexes were seen during the stretch, consistent with a Valsalva manoeuvre, and slowing of the EEG rhythms into the theta range coincided with his symptoms 5 seconds later. If loss of consciousness ensued, then earlier and more marked slowing of the EEG into the delta range was seen just two seconds after stretching, and these patterns are consistent with the nonspecific delta/theta slowing recorded elsewhere during tilt table-induced syncopal episodes [8] which likely reflect widespread cerebral dysfunction. No comment is made about the heart rate during this time and intra-arterial blood pressure analysis was not done. Again, baseline brain MRI, extracranial Doppler ultrasound, ECG, EEG (waking and sleep), and tilt table tests were all normal. Also normal was their response to a carotid sinus massage which would suggest that the head turning manoeuvre itself was not mechanically stimulating a hypersensitive carotid baroreceptor. In any case, reduced blood flow within the vertebrobasilar circulation alone in young patients with normal intracranial vessels, otherwise, should not necessarily precipitate a loss of consciousness and implies a more global cerebral hypoperfusion.

The suggestion that SSA arose solely through a dysfunction of the posterior circulation was, therefore, challenged following the publication of further cases in which EEG video telemetry, intra-arterial blood pressure measurement, and transcranial Doppler of middle cerebral artery (MCA) flow were recorded in three patients [3]. Towards the end of a stretch, but not an isolated Valsalva, sinus tachycardia was repeatedly seen accompanied by a systemic hypotension and MCA diastolic flow arrest. Two of the patients experienced a complete loss of consciousness and the third a decreased awareness, with the EEG simultaneously demonstrating generalised slow waves in each. Thus, it was proposed that SSA is a unique form of reflex syncope with tachycardia and systemic hypotension, as opposed to vasovagal syncope and carotid sinus hypersensitivity where hypotension is accompanied by bradycardia.

In summary, the true prevalence of stretch syncope is unknown, but the adolescent preponderance of reported cases might suggest that it is a phenomenon restricted to the young. Patients may, therefore, present to general practitioners, emergency departments, and both paediatric and adult neurology services with their episodes of collapse. Apart from one case where the blood pressure responses were abnormal during a Valsalva manoeuvre (although remaining insufficient to induce syncope on its own) [5], the battery of neurological and cardiac investigations between attacks are largely unremarkable [1, 3–5]. Distinction from temporal seizures may be tricky if altered consciousness states arise, as noted in one case above, but the “ictal” EEG showed no epileptiform activity [3]. The key, as ever, is in the history with each episode preceded, sometimes willfully, by an act of stretching. Once our patient was advised to avoid the provoking manoeuvre, her blackouts ceased.
